# The transportability of Memory Specificity Training (MeST): adapting an intervention derived from experimental psychology to routine clinical practices

**DOI:** 10.1186/s40359-019-0279-y

**Published:** 2019-02-01

**Authors:** Kris Martens, Tom J. Barry, Keisuke Takano, Filip Raes

**Affiliations:** 1 Faculty of Psychology and Educational Science, KU Leuven, Tiensestraat 102, 3000 Leuven, Belgium; 2 Department of Psychology, The University of Hong Kong, Jockey Club Tower, Pokfulam Road, Hong Kong, Hong Kong; 3 Department of Psychology, The Institute of Psychiatry, King’s College London, BOX PO77, Henry Wellcome Building, De Crespigny Park, Denmark Hill, London, SE5 8AF UK; 4 Department of Psychology, Ludwig-Maximilians-University of Munich, Leopoldstrasse 13, 80802 Munich, Germany

**Keywords:** Memory specificity training, Autobiographical memory, Adaptability, Transportability

## Abstract

**Background:**

Accumulating evidence shows that a cognitive factor associated with a worsening of depressive symptoms amongst people with and without diagnoses of depression – reduced Autobiographical Memory (rAMS) – can be ameliorated by a group cognitive training protocol referred to as Memory Specificity Training (MeST). When transporting interventions such as MeST from research to routine clinical practices (RCPs), modifications are inevitable, with potentially a decrease in effectiveness, so called *voltage drop.* We examined the transportability of MeST to RCPs as an add-on to treatment as usual with depressed in- and out- patients.

**Methods:**

We examined whether 1) MeST was adaptable to local needs of RCPs by implementing MeST in a joint decision-making process in seven Belgian RCPs 2) without losing its effect on rAMS. The effectiveness of MeST was measured by pre- and post- intervention measurements of memory specificity.

**Results:**

Adaptations were made to the MeST protocol to optimize the fit with RCPs. Local needs of RCPs were met by dismantling MeST into different subparts. By dismantling it in this way, we were able to address several challenges raised by clinicians. In particular, multidisciplinary teams could divide the workload across different team members and, for the open version of MeST, the intervention could be offered continuously with tailored dosing per patient. Both closed and open versions of MeST, with or without peripheral components, and delivered by health professionals with different backgrounds, resulted in a significant increase in memory specificity for depressed in- and out- patients in RCPs.

**Conclusions:**

MeST is shown to be a transportable and adaptable add-on intervention which effectively maintains its core mechanism when delivered in RCPs.

**Trial registration:**

ISRCTN registry, IDISRCTN10144349, registered on January 22, 2019. Retrospectively registered.

**Electronic supplementary material:**

The online version of this article (10.1186/s40359-019-0279-y) contains supplementary material, which is available to authorized users.

## Background

Although multiple empirically supported treatments are available [1], recent studies suggest that the effects of existing psychotherapies for depression may be smaller than first thought and may be overestimated [2]. Two routes have been suggested by which the efficacy of treatments might be improved: linking particular *mechanisms* with specific interventions [3] and improving the *implementation* of interventions [4]. The current study takes both of these approaches and examines the transportability of an add-on intervention derived from experimental research targeting one specific cognitive factor: difficulty retrieving specific, personal memories of events lasting less than a day. This phenomenon is referred to as reduced Autobiographical Memory Specificity (rAMS) or Overgeneral Autobiographical Memory (OGM) [5]. rAMS is regarded as clinically relevant due to its association with a range of maladaptive psychological processes and outcomes, for example increases in repetitive negative thinking [6, 7], hopelessness [8, 9] and problem solving deficits [8, 10–12]. Through its effects on these intervening outcomes rAMS is considered to be an enduring trait of depression [5, 13, 14].

Given its contribution to depression, rAMS is a promising phenomenon to target with an intervention. To that end, Memory Specificity Training (MeST) was developed and was initially tested in an uncontrolled clinical trial in depressed people [15]. MeST was found to lead to an increase in memory specificity and improvements in associated cognitive processes (problem solving, rumination and hopelessness) in 10 depressed female inpatients. Although many existing MeST trials have methodological limitations and do not always show positive effects on symptomatology, subsequent investigations with MeST have shown positive results indicating that memory specificity is modifiable and such modification in turn affects the symptoms of emotional disorders and other cognitive processes (such as rumination) that mediate the association between rAMS and emotional disorder [16–20].

As evidence accumulates in research contexts, the question of whether MeST is transportable to routine clinical practice (RCP) arises. In this context, we refer to RCPs as clinical settings with less resources than those involved in research and in which the principle care providers are health professionals with a variety of professional backgrounds, often with a higher clinical load, and less expert supervision than might be the case in research settings [4]. Transportability (or transferability) can be defined as “the degree to which treatments that demonstrate efficacy in controlled research designs can be utilized in [non-research] settings with similar benefit” ([21], p946). In this study we operationalize transportability of MeST as being adaptable to local needs of RCPs whilst achieving similar benefit in increasing memory specificity as the original research-based MeST program.

When transporting interventions such as MeST from research to RCPs, modifications are inevitable, with potentially negative consequences for the effectiveness of the intervention, so called *voltage drop* [22]. As possible modifications to interventions during implementation can vary in many ways, Stirman and colleagues [23] designed a system for classifying such modifications, based on five questions: (a) by whom are modifications made; (b) what is modified (content, context or training and evaluation); (c) at what level of delivery were modifications made (e.g. individual patient level, hospital level, …); (d) if context modifications are made, to which part are they made (format, setting, personnel, population); and, (e) what is the nature of the content modifications? For the current study, our main focus as researchers mainly involved in efficacy research was which modifications to the intervention (MeST) were needed, with the goal of evaluating the transportability of MeST for all future implementations made by settings or implementation researchers. For this to be the case, we implemented MeST in a variety of contexts: with inpatients and outpatients, general and psychiatric hospitals, MeST being part of a full therapy schedule or not, and with different age groups (adults and elderly).

As we focused on adaptations made to MeST, the domain *Characteristics of the intervention* of the Consolidated Framework For Implementation Research (CFIR) [24] offered a useful list of constructs to be considered during the dissemination and implementation of the program. The other four domains (*Inner Setting*, *Outer Setting*, *Individuals involved* and the *Implementation Process*) are less under the influence of researchers involved in efficacy research, but were nonetheless important given their potential impact on other aspects of the implementation process. The eight constructs of the CFIR domain *Characteristics of the Intervention* are *Intervention Source*, *Evidence Strength and Quality*, *Relative Advantage*, *Adaptability*, *Trialability*, *Complexity*, *Design Quality* and *Cos*t (definitions can be found in Table 1).

**Table 1:** Eight constructs of the characteristics of the intervention of the consolidated framework for implementation research (CFIR) ([22], “Results”, para 2–8)

Construct	Definition
Intervention Source	“Perception of key stakeholders about whether the intervention is externally or internally developed” (…) “The legitimacy of the source may also influence implementation”
Evidence Strength and Quality	“Stakeholders’ perceptions of the quality and validity of evidence supporting the belief that the intervention will have desired outcomes”
Relative Advantage	“Stakeholders’ perception of the advantage of implementing the intervention versus an alternative solution”
Adaptability	“The degree to which an intervention can be adapted, tailored, refined, or reinvented to meet local needs”
Trialability	“The ability to test the intervention on a small scale in the organization and to be able to reverse course if warranted”
Complexity	“Perceived difficulty of implementation, reflected by duration, scope, radicalness, disruptiveness, centrality and intricacy and number of steps required to implement”
Design Quality	“Perceived excellence in how the intervention is bundled, presented and assembled”
Costs	“Costs of the intervention and costs associated with implementing that intervention, including investment, supply, and opportunity costs”

Of these constructs, Adaptability fits our research question the most – that is, to what degree is the MeST protocol adaptable to CRPs. An extra distinction for this construct is made between ‘*Core Components*’, the essential elements of the intervention, and ‘*Adaptable Periphery*’, the non-essential and modifiable elements of the intervention package. The core component of MeST is the retrieval of specific memories and providing details of those memories by answering follow-up questions, conducted during sessions and as home work assignments. However, MeST also includes several secondary components which make up its adaptable periphery, such as psycho-education regarding memory problems linked to depression and a psycho-education and exercises on a model (STOP-model) to notice overgeneral thinking. An additional file describes MeST comprehensively [see Additional file 1]. In total, participants are offered 104 cue word exercises in this version of MeST. In subsequent studies a fifth [16, 25] and sixth [26] session were introduced. An implicit assumption underlying these adaptions is that more exercises are required, although this assumption has not been tested.

Other broader characteristics of the training are that a) MeST happens in group, b) with a trainer and c) exercises are increasingly more challenging. In the original MeST, the degree of difficulty increased throughout the training by increasing the amount of exercises per session, by changing the valence of the cue words (negative cue words are introduced in Session 3) and by adding more complex exercises later in the training. The internal logic of MeST is that greater practice in retrieving specific memories will be associated with greater improvement in rAMS.

In searching for the right balance between fidelity to the core component of an intervention and adaptability to RCP, Backer [27] proposed six steps to maintain this balance. Following these steps, the present investigation adapts MeST according to the local needs of seven Belgian RCPs. The two first steps, identifying and understanding the theory base behind the program (Step 1) and locating core components of the program (Step 2), are clear for MeST as it is a theory driven intervention emerging from the basic science of rAMS. Other steps, involve addressing fidelity/adaptation concerns amongst clinicians (Step 3), consulting with the intervention developer if needed (Step 4), and consulting each organization involved in the implementation (Step 5). An implementation plan based on these inputs and experiences of settings can then be developed (Step 6). This present investigation follows this stepped process of joint decision-making for *each* RCP setting.

In summary, we examined the adaptability of MeST by implementing it in several clinical settings, conducting adaptations based on local needs of RCPs in different contexts, and examined whether these adapted versions continued to target the core mechanism of MeST, reduced memory specificity. Adaptability was examined by checking if local needs which arose during implementation were met, measured in terms of anecdotal feedback given by local clinicians, while examining if adapted versions of MeST still influenced the core mechanism by increasing memory specificity from pre- to post- intervention (quantitative evaluation). Failure to meet local needs through the adaptation process would mean that MeST had limited adaptability. If local needs were successfully met but the adapted version failed to improve memory specificity, then MeST’s adaptability would be limited and an evaluation of MeST’s core mechanism would be needed. If local needs were successfully met and the adapted versions demonstrated a significant improvement in memory specificity then this would support the conclusion that MeST possessed high adaptability.

## Methods

### Participants – Settings and patients

Before this project started, one residential psychiatric hospital already implemented MeST on its own initiative. This setting was included in the present study and feedback on the implementation process of this setting at the start of this study had an important influence on how the basic protocol was adapted. The other participating settings were one outpatient treatment setting affiliated with the University of the first and last author, two outpatient and one inpatient psychiatric setting both of which were part of a general hospital, and three inpatient wards of residential psychiatric hospitals. The settings and participating patients of each setting are described in Table 2.

**Table 2:** Description of settings and patients

Setting	1	2	3	4	5	6	7	Total
Name	PraxisP	Asster	Sophia (PZ Duffel)	Fase 4 (PZ Duffel)	Jessa (PAAZ)	Sint Franciscus Ziekenhuis Heusden Zolder (PAAZ)	Algemeen Stedelijk Ziekenhuis Aalst (PAAZ)	
Patient Population	Outpatients – depressed adults	Inpatients – depressed adults	Inpatients – depressed older adults	Inpatients – depressed adults	Outpatients – depressed adults	Outpatients – depressed adults	Inpatients – depressed adults	
Timing	March 2015 – April 2015	March 2015 – January 2018	March 2015 – January 2018	March 2015 – January 2018	April 2015 – June 2015	February 2015 – April 2015	March 2015 – June 2015	
Intakes	7	52	98	143	10	12	6	328
Eligibility Criterion	Yes, AMT < 70%	Yes, AMT ≤ 70%	No.	Yes, AMT ≤ 70%	No.	Yes, AMT ≤ 70%	Yes, AMT < 70%	
Eligible for Training	5	36	98	66	10	10	4	229
Drop out before post measurement	0	24	31	45	3	2	2	107
Pre- and Post- measurements available	5	12	66	21	7	8	2	121

### Measures

#### Autobiographical memory specificity

Autobiographical Memory Specificity was measured pre- and post- training using two sets of cues of an oral version of the Autobiographical Memory Test (AMT) [28]. Patients were orally instructed to retrieve a specific memory for each of 10 cue words (five positive, five negative) presented. The instructions included that the memory needed to be specific, happened once and lasted shorter than a day but did not have to be an important event. Within the instructions, after examples of specific and non-specific responses were given, a practice trial with three cue words with feedback took place. Throughout the test non-specific and unclear answers were followed with prompts until patients succeeded in retrieving a specific answer or until 1 minute passed. The AMT was scored as the number of first answers that were coded as specific.

#### Depressive symptomatology

Settings were offered the possibility to measure self-reported depressive symptomatology pre- and post- intervention with the Patient Health Questionnaire 9 (PHQ-9) [29] or the Beck Depression Inventory II (BDI-II) [30, 31]. The PHQ-9 contains nine items of depressive symptoms which refers to DSM-IV depression diagnostic criteria and other leading Major Depressive Disorder symptoms. Scores can vary from 0 to 27. The BDI-II assesses severity of depressive symptoms and consists of 21 questions, scores vary from 0 to 63. For both the PHQ-9 and BDI-II patients are asked to mark the statements that best describe how they felt during the past 2 weeks. Both questionnaires have shown good internal consistency with Cronbach’s alphas ranging from .86 to .89 for PHQ-9 [32] and from 0.83 to 0.96 for the BDI-II [33].

#### Rumination

The Ruminative Response Scale – Brooding subscale (RRS-Brooding) [34, 35] is a self-report questionnaire consisting of five items, part of the 22 items of the Ruminative Response Scale [36], measuring brooding. The items on the brooding factor are considered to measure the maladaptive coping of passively comparing one’s situation with some unachieved standard. Patients are asked to report how frequently they tend to think of certain thoughts on a 1 (*almost never*) to 4 (*always*) scale. Scores vary from 5 to 20. Internal consistency is considered acceptable, with a Cronbach’s alpha of .76 [37].

#### Memory specificity training (MeST)

Original materials and guidelines from the first MeST study [15] were used. Session 1 of MeST focuses on psycho-education regarding memory problems linked to depression. Three main memory problems are described: reduced levels of concentration, a bias in retrieving mainly negative memories and rAMS. It is explained to participants, within a group setting, that only rAMS can be considered as a risk factor for depression and that training can remediate rAMS to some extent. After this psycho-education, two specificity exercises are conducted within the group. Exercises consist of a presented cue word after which participants are encouraged to retrieve a specific memory and as many details as possible. After each participant writes down their memory, participants help each other with becoming more specific by asking for more details. The session ends by introducing a homework assignment: to re-read the psycho-education, to write down one specific memory for each of 10 (positive & neutral) words and to write down one memory of the day at the end of each day.

In Session 2, after briefly repeating the psycho-education, homework assignments are discussed. Next, some exercises are conducted together within the group wherein participants need to retrieve two memories for one cue word. Homework assignments after this session consist of writing down two memories for each of 10 (neutral & positive) cue words and writing down two memories of the day each day.

Session 3 has a similar structure but the exercises now offer word pairs of two opposing valences (e.g. skilful and clumsy). The homework assignment contains two memories for each of 10 words (neutral, positive but also negative) and writing down two memories of the day each day. In the fourth and last session, after evaluating the homework assignments, a psycho-education on the STOP-model is given. The aim here is that participants learn to notice when they are overgeneralizing by: signalling to themselves when they are thinking at an overgeneral level; trying to think back to the specific event that prompted the overgeneral thinking; to obtain and generate specific details about that event as much as possible; and, as a last step, try to find an opposite example. After this, some more exercises with opposing cue words are conducted.

In subsequent studies a fifth [16, 25] and sixth session [26] were introduced where participants were invited to conduct exercises based on the STOP-model; they were instructed to think back and write down when they were thinking at an overgeneral level, describe which specific event prompted the overgeneral thinking (as detailed as possible) and come up with and write down an opposite example. Two other new elements were added: a revision and take-home-message in the last session and in the last session homework exercises are provided so participants can continue to train further.

To adapt MeST to Routine Clinical Practices, the six variations of specificity exercises (for example, one memory for one cue word, or two memories for a word pair) were converted to six different instruction forms, creating the opportunity for patients and trainers to choose together how many exercises they wish to conduct and to choose the level of difficulty of each homework assignments (e.g., two memories for one cue being more difficult than one memory per cue). All possible cue words were put in a list so that combinations between instructions forms and cue words could be made.

#### Other measures

Settings were invited to keep track of the amount of sessions and exercises completed per participant.

### Procedure

The first author first ran MeST in a setting affiliated with KU Leuven (setting 1), for which participants were recruited via the newsletter (e-mail) of the setting and via an e-mail to clinical psychologists in the region. This training was framed as a study on the transportability of MeST and was offered free of charge. Settings 3 and 5 contacted the last author asking for information about new developments in treating depression, after which they were invited to participate in this study. Setting 4 and 6 showed interest in participating after hearing about the study from setting 3 and 5. Setting 7 showed interest in participating after hearing about the study from the first author. In a next step, the first author visited these settings and presented the principles of MeST in a team meeting. From there on, a joint discussion-making process was initiated and adjustments were made to the protocol. Decisions about how and when to start the training where made by phone and e-mail. The first author conducted the first one to three assessments of participants and first one to three sessions in settings 2 to 7, while trainers of the settings co-operated and were able to continue the assessment and MeST independently. In most cases, the trainers of settings conducted some assessments and sessions in the presence of the first author, so that feedback was provided. All participants were patients who were assigned to the therapy program of each setting, except for Setting 1 where patients exclusively participated in MeST.

For each patient involved, a pre-intervention measurement of rAMS was conducted after which patients were invited to participate in MeST. In setting 2 MeST was already running but no pre- and post- intervention measurements of memory specificity were being conducted. To be included in this study, setting 2 started using pre- and post- intervention measurements and Informed Consent forms.

We advised settings to only include patients who experienced rAMS, which was operationalized as recall of fewer than 70% specific memories, relative to the total number of cue words in the AMT. Also, settings were instructed to counterbalance the two cue sets (AMT A and AMT B) between pre- and post- intervention measurement. The study received institutional ethical approval of the Social and Societal Ethics Committee of the University of Leuven and all patients filled out and signed an Informed Consent form.

## Results

### Evaluation of the feasibility of MeST and adaptations made

#### Challenges for the implementation of MeST

First, we report findings based on anecdotal feedback about the challenges faced whilst implementing MeST in RCPs and the adaptations that were made. In setting 2 MeST was already implemented at the setting’s own initiative and feedback on the implementation process of this setting at the start of this study had an important influence on how the basic protocol was adapted. This feedback from the various settings regarding the challenges of delivering MeST fell within several themes 1) the nature of treatment within settings where patients are constantly transitioning through the services; 2) dosage; 3) patient motivation; and, 4) the nature of treatment delivery by multidisciplinary teams.

The first and probably most important factor that arose during the implementation phase was the continuous admission and discharge of patients. In research protocols the training is offered in a *closed* format with a fixed set of sessions, but this format was regarded as impractical for some RCPs. Patients are admitted and dismissed continuously in hospitals, so the risk of dropout or not being able to participate in MeST was high.

Second, questions of dosage were raised. It was unclear to RCPs and to us as program developers how much training was necessary within each setting. According to the clinicians involved, four sessions were considered as too limited as most patients did not conduct ‘sufficient’ homework assignments. Also, the number of exercises per and between sessions was considered too demanding. We concluded, based on the received feedback, that the amount of exercises within the original MeST program was hard to achieve for patients in most RCPs.

This was associated with the third challenge; the motivation of patients in RCPs. Patients in RCPs conducted less exercises and settings reported that patients were not motivated to conduct the recommended amount of exercises. Patients in RCPs did not consciously choose which therapy program they participated in (in comparison to participants engaging in typical research studies) and are often included in a more challenging therapy program.

The fourth challenge (which can be regarded as an opportunity as well) was the multidisciplinary nature of all RCPs (with the exception of the university-aligned centre, setting 1). In setting 2 at the start of this project, for example, homework assignments of inpatients were not followed up by other team members because they were insufficiently informed about the content of the training.

#### Adaptations of MeST

To answer the given four challenges, we dismantled MeST into different subparts. One benefit of dismantling MeST was that different components could be conducted by different team members, which makes the burden of implementing MeST for such multidisciplinary settings less challenging. Table 3 shows in which parts the MeST protocol was dismantled and how tasks were divided over different disciplines with different professional and educational backgrounds. Dismantling MeST in different settings resulted in two different forms of MeST; the *closed* version similar to the research protocol, and an *open* version in which patients were able to join and stop the training whenever they wished to and in which each participants received tailored homework exercises.

**Table 3:** Dismantled version of MeST and which discipline performs which part in each setting

Setting	1	2	3	4	5	6	7
Open or closed version?	Closed	First closed, than open	Open	Open	Closed	Closed	Closed
Eligibility & Pre-measurement Phase							
Deciding on eligibility	Clinical Psychologist	Team	Team	Team	Team	Team	Team
Pre-measurement: AMT	Clinical Psychologist	Nurse	Nurse	Occupational Therapist	Clinical Psychologist	Clinical Psychologist	Clinical Psychologist
Pre-measurement: depressive symptoms	Clinical Psychologist	Nurse	Nurse	Clinical Psychologist	Clinical Psychologist	Clinical Psychologist	Clinical Psychologist
Informed Consent	Clinical Psychologist	Nurse	Nurse	Occupational Therapist	Clinical Psychologist	Clinical Psychologist	Clinical Psychologist
Training Phase							
Psycho-education	Clinical Psychologist	Nurse	Nurse	Occupational Therapist	Occupational Therapist	Clinical Psychologist	Clinical Psychologist
Specificity Exercises	Clinical Psychologist	Nurse	Nurse	Occupational Therapist	Occupational Therapist	Clinical Psychologist	Clinical Psychologist
STOP model	Clinical Psychologist	Nurse	Clinical Psychologist	Occupational Therapist	Occupational Therapist	Clinical Psychologist	Clinical Psychologist
Post-measurement & Last Phase							
Post-measurement: AMT	Clinical Psychologist	Nurse	Nurse	Occupational Therapist	Clinical Psychologist	Clinical Psychologist	Clinical Psychologist
Post-measurement: depressive symptoms:	Clinical Psychologist	Nurse	Nurse	Clinical Psychologist	Clinical Psychologist	Clinical Psychologist	Clinical Psychologist
Revision	Clinical Psychologist	Nurse	Nurse	Occupational Therapist	Occupational Therapist	Clinical Psychologist	Clinical Psychologist

#### An open version of MeST - advantages and disadvantages

Advantages of the open version experienced by RCPs can be summed up as a) more tailored to individual patient needs and the constraints of a given setting; b) less risk of drop-out; c) increased possibility of installing MeST as a continuous part of a therapy program by which more patients could be trained in future; d) greater ease of getting back involved in the training if a patient dropped out; e) the STOP-session could be repeated more often than would otherwise be the case in the normal research protocol, so patients were offered more opportunities to generalize the trained skill.

Disadvantages of the open version were that a) clinicians felt that patients completed less exercises; b) the dropping in and out of patients created a less safe environment to share personal memories; c) making it tailored to each patient demanded more effort for therapists in deciding what, and how many, homework assignments to assign, preparing the sessions practically (printing materials) and discussing homework assignments as patients conducted different exercises, d) having an endless continuous cycle of similar sessions risked being monotonous for the therapist. Another advantage of having two versions of MeST available to RCPs was that settings could first try out a closed version, after which switching to an open version was a possibility, which increased Trialability (CFIR-construct, see Table 1) of MeST.

#### Feedback of the settings

Settings also gave broader feedback on MeST and its implementation in RCP settings. Clinicians raised issues regarding the inclusion criteria. MeST is an add-on intervention targeting a risk factor. However, the reality in the timeframe of this study in Belgium was that most settings applied a general diagnosis-treatment model in organizing health care. Running a time-consuming pre-intervention measurement of a risk factor for each patient and designing tailor-made therapy programs for each patient was not compatible with the RCPs involved at the time of the study. The AMT was experienced as time consuming by trainers.

Settings also reported that the (emotional) cue words for the pre-intervention AMT measurement were upsetting some patients. Although MeST started with neutral cue-words, the AMT did not. Some trainers said that this reduced patients’ motivations for participants in the study. There were also problems using an informed consent form. Due to the experimental stage MeST was in while this project ran we had to get participants’ consent. For some patients the fact that for one session of their therapy program they needed to sign such a form was confusing.

A consequence of our assessment of rAMS prior to MeST, and thus excluding patients who did not meet the threshold criterion, was that not all patients could (or should) participate. In combination with the constant transitioning of patients plus some patients who did not want to start after conducting the AMT, some settings struggled to include enough participants to consider it worth the time investment. As a result, some settings decided to deviate from the recommended eligibility criterion throughout the implementation process.

Trainers also fed back that cue-words were sometimes too difficult for the given patient population, and the take-home message was often perceived as too complex. The length of a session in the closed version was a fifth concern raised by trainers. Some trainers considered 60 min too short to complete enough exercises. A sixth concern was that the STOP-model was difficult to train and challenging to understand for patients. This resulted in setting 3 excluding the STOP-model during the study.

In conclusion, the main needs of RCPs were met by the adaptations made, however, several additional challenges remain.

### Quantitative analysis of MeST’s effects

Second, we report quantitative data regarding the impact of the training on the core mechanism of MeST and on depressive symptomatology and rumination. The results on depressive symptomatology and rumination should be interpreted with caution as only setting 1 offered patients MeST exclusively; all other settings offered MeST as part of a full time therapeutic program. Reductions in depressive symptoms and rumination are given as an indication of the improvement in the symptomatology of the participants. The relations with dose and amount of exercises completed were also examined.

#### Descriptive statistics: Eligibility, sessions and exercises completed

Due to the fact that some settings chose to deviate from the recommended inclusion criterion, these criterion varied between settings between a) no criterion, b) scoring less *or equal to* 70% on memory specificity within the AMT, or c) scoring *less than* 70% (as recommended). In sum, pre- and post- intervention measurement data was available for 121 patients (Table 2; 52.8% of participants). Some settings collected data on how many sessions and exercises patients conducted; between 1 and 11 sessions with a mean of 20 exercises (*SD* = 12.21) completed. Table 4 describes for each setting how many patients’ data were collected, and the minimum, maximum, mean and standard deviations of the amount of sessions they participated in and exercises they completed.

**Table 4:** For four settings and in total: Sessions participated and exercises completed

Setting	1	2	3	4	Total
Number of patients of which number of sessions is known	5	6	97	34	142
Minimum number of sessions	5	3	1	1	1
Maximum number of sessions	5	10	8	11	11
*M* (SD)	5.00 (0.00)	5.50 (2.35)	5.42 (2.42)	5.94 (2.63)	5.54 (2.42)
Number of patients of which number of completed exercises is known:	/	6	34	33	73
Minimum number of exercises	/	5	0	2	0
Maximum number of exercises	/	76	36	35	76
*M* (SD)	/	36.33 (23.37)	21.53 (9.58)	15.33 (8.98)	19.95 (12.21)

#### Core mechanism: Increasing memory specificity

The adapted versions of MeST were associated with effectively increasing memory specificity in each setting, with the exception of one setting which only provided pre- and post- intervention measurements of two participants. Table 5 shows results for each setting, and the overall results across settings. Mean scores (*n* = 121) increased significantly from 4.76 (*SD* = 2.07) at pre-intervention to 7.46 (*SD* = 2.03) at post-intervention, *t*(120) = 12.96; *p* < .001. Using a mean of pre-post difference scores and a 95% CI using a pooled SD of pre-intervention scores this results in a mean difference of 2.70, 95% CI [1.90–3.50].

**Table 5:** Pre and post-training measurement of memory specificity (AMT) for each setting and in total

Setting	1	2	3	4	5	6	7	Total
AMT pre *M* (*SD*)	4.40 (1.67)	3.75 (1.96)	5.02 (1.87)	4.14 (2.22)	7.29 (2.22)	4.13 (1.96)	3.50 (.71)	4.76 (2.07)
AMT post *M* (*SD*)	7.80 (.45)	7.17 (2.59)	7.35 (2.03)	7.38 (1.69)	9.86 (.38)	7.38 (2.39)	5.00 (.00)	7.46 (2.03)
*t*	4.54	5.86	8.14	5.78	3.58	3.87	3.00	12.96
*p*	.010	<.001	<.001	<.001	.012	.006	.205	<.001
Mean difference*,* 95% CI	3.40	3.42	2.33	3.24	2.57	3.25	1.50	2.70 [1.90–3.50]
N	5	12	66	21	7	8	2	121

*Note*. *AMT* Autobiographical Memory Test. A mean of pre-post difference scores is calculated per setting. A 95% CI is calculated for the total sample by using a pooled SD of the pre-intervention scores

To exclude the possibility that a decrease in depressive symptoms explained the increase in memory specificity, a repeated measures ANCOVA was run, using standardized residual change scores of depressive symptoms as a covariate. Changes in memory specificity remained significant even after controlling for changes in depressive symptoms, *F*(1, 17) = 45.08, *p* < .001 for 19 participants whose PHQ-9 change scores were available; and *F*(1, 17) = 44.58, *p* < .001 for 19 participants whose BDI II change scores were available. Participants’ memory specificity improved from pre- to post-intervention and this was independent of any changes in depressive symptoms.

#### Dose-effect relation

No significant association was found between the amount of exercises completed and the size of the increase in memory specificity between pre- and post- measurement (*n* = 54, *r* = .16, CI 95% [−.11–.41], *p* = .24).

#### Depressive symptoms and rumination

When all settings were considered together, overall participants showed a significant decrease in self-reported depressive symptoms and rumination from pre- to post- intervention (Table 6). It is of note that in Setting 1, in which the group patients received MeST as a stand-alone intervention (*n* = 5), no significant impact of MeST of each of the measures was found (see Table 6).

**Table 6:** Depressive symptoms (PHQ-9 and BDI II) and Rumination (RRS-5) for each setting and in total

Setting	1	2	4	5	6	7	Total
Depressive symptoms – PHQ 9							
Pre-, M (SD)	12.00 (5.61)	14.10 (4.41)		12.40 (5.27)			13.15 (4.76)
Post-, M (SD)	7.60 (5.03)	9.50 (2.59)		6.20 (7.23)			8.20 (4.64)
*t*	1.45	3.11		4.70			4.67
*p*	.22	.01		.01			<.001
Mean difference, 95% CI	−4.40	−4.60		−6.20			4.95 [2.86–7.04]
n	5	10		5			20
Depressive symptoms – BDI II							
Pre-, M (SD)	26.80 (5.40)		29.00 (11.72)		34.40 (7.02)	34.50 (2.12)	30.42 (8.61)
Post-, M (SD)	20.60 (11.39)		25.00 (12.49)		21.80 (6.38)	29.00 (1.41)	23.42 (9.87)
*t*	1.64		1.13		2.90	2.20	3.42
*p*	.18		.30		.04	.27	.003
Mean difference, 95% CI	− 6.20		−4.00		−12.60	−5.50	7.00 [3.13–10.87]
n	5		7		5	2	19
Rumination – RRS – Brooding							
Pre-, M (SD)	13.60 (3.58)	12.80 (3.12)					13.07 (3.17)
Post-, M (SD)	11.40 (2.08)	10.70 (1.95)					10.93 (1.94)
*t*	1.33	2.51					2.85
*p*	.26	.03					.013
Mean difference, 95% CI	−2.20	−2.10					2.13 [.52–3.74]
n	5	10					15

Note. *PHQ-9* Patient Health Questionnaire 9, *BDI II* Beck Depression Inventory II, *RRS Brooding* Ruminative Response Scale. A mean of pre-post difference scores is calculated per setting. A 95% CI is calculated for the total sample by using a pooled SD of the pre-intervention scores

For the 14 participants for which rumination (RRS-Brooding) and memory specificity data were available, contrary to our expectations, standardized residual change scores for rumination and specificity across treatment did not correlate significantly (*r* = −.02, CI 95% [−.54 .52], *p* = .95).

## Discussion

MeST is a group training protocol which targets a risk factor associated with depression, reduced autobiographical memory specificity. Research to-date suggests that MeST may hold promise as an intervention within depression. In particular, MeST ameliorates rAMS and affects associated symptoms and psychological processes [15, 16, 20]. However, until now, MeST has been confined to research settings and for its potential clinical utility to be realised, it is critical to demonstrate that it has comparable effects when transported to routine clinical practices where depressed patients in the community would typically access treatment. The goal of this study was to evaluate and develop the transportability of MeST by implementing MeST in, and adapting MeST to, diverse clinical settings. The main focus was on finding a balance between fidelity to the core of MeST and adapting the characteristics of the intervention to the needs of RCPs. The results of this study show that MeST is adaptable to the local needs of RCPs while still being effective in increasing memory specificity. As an answer to the four main challenges that arose during implementation (patients constantly transitioning through the services, dosage, patient motivation and multidisciplinary nature of RCPs), dismantling MeST into different subparts created several opportunities. First, different team members were able to take up different parts of the MeST procedure. MeST was administered in different settings by trainers with different professional backgrounds (nurses, occupational therapists, clinical psychologists). Second, an open version was created that enabled continuous provision even when participants missed treatment sessions or dropped out for extended periods. Third, an open version offered patients the possibility to train at their own pace, and dosage could be tailored to each patient. Offering the core concepts of a treatment in a free standing treatment session – *modularizing* – is considered as one of the steps forward in the roadmap to adapting ESTs successfully according to Strosahl and Robinson [38].

Our quantitative analyses showed that all adaptations of MeST increased memory specificity significantly, suggesting that adapted versions are still effective in modifying the core mechanism. Comparing the current effect (a mean difference of 2.70 on the AMT, 95% CI [1.90–3.50]) with a previous study with high internal validity which used the same inclusion criterion [20] in which participants increased from a mean of 5.2 (SE = 0.4) to a mean of 8.0 (SE = 0.4), shows that the adaptations made for RCPs here did not decrease the efficacy of MeST in a significant way. Also, a translation to an open version (with a mean difference ranging from 2.33 to 3.42 in Settings 1, 2 and 3; see Table 5) showed comparably strong effects. The phenomenon of *voltage drop* [22] does not seem to have occurred.

These results also indicate that a lower dosage does not necessarily compromise MeST’s effectiveness. The original MeST protocol [15] contained 4 sessions and 104 specificity exercises, all subsequent studies increased the amount of sessions and exercises. In this study the mean number of completed exercises was lower (*M =* 19.95, *SD =* 12.21) due to differences between RCPs and research settings such as less functioning and less motivated patients, and MeST often being part of full time therapy programs. Our results indicate that adapted MeST with a lower amount of sessions still increases memory specificity.

Because the first author went to educate each team about the content of MeST, this probably had a potential positive influence on the implementation process. Multidisciplinary teams in residential settings require information about how each team member can contribute to increasing autobiographical memory specificity. Future MeST protocols can include a standardized psycho-education for teams. As some settings considered the pre-intervention AMT assessment as being too time consuming, a second possible adaptation for future MeST protocols would be to start using the computerized scoring algorithm [39] as an automated assessment. Having an automated version of the AMT which scores specificity of patients’ memories automatically, without the need for experimenters or clinicians to judge the specificity of each memory could save time in ensuring that adapted MeST protocols will only be provided for patients who experience rAMS. In addition, an automatized AMT heightens the possibility that RCPs track improvements of patients, standardizes delivery across sites and reduces the burden placed on clinicians.

In the coding system to classify modifications in implementation by Stirman and colleagues [23], the modifications in this study would be coded as tailoring/tweaking refining (creating slightly different versions of handouts for the open version), removing elements (STOP model), shortening/condensing (the amount of exercises), lengthening/extending (the amount of sessions in the open version) and thus ‘loosening the structure’, and finally ‘repeating elements’ (in open sessions participants can train the same kind of cue words again and again). The MeST manuals used during implementation included very detailed therapist instructions, along with detailed handouts and worksheets to guide each session. Future MeST protocols should combine the open and closed manuals in one format and in doing so offer settings the possibility to choose how to implement MeST. An important barrier to the dissemination of any psychological treatment is the difficulty in finding the necessary funding to train clinicians [40]. The amount of training in this study was limited; the first author went to team meetings to introduce MeST and after discussing implementation issues and deciding about how and when the training started, the first author modelled a few assessments and sessions to the local trainers. In most cases, roles were reversed to give some feedback to trainers. The current results therefore suggest that MeST is an intervention that is low in *Complexity* (CFIR construct, see Table 1). One possible future research avenue would be to design a train-the-trainer protocol which includes all information and materials necessary to implement MeST in any given setting.

While this study has obvious implications for the application of MeST in routine clinical settings, more broadly this study also illustrates the added value of implementation science for researchers involved in efficacy research. Examining adaptability early in the development of an intervention – in this case before the quality of evidence was considered as a strong recommended intervention according to the GRADE system [1] – can be beneficial. If this study resulted in implementation failures this could have impacted the potential interest in subsequent efficacy studies with MeST. Conducting such an adaptability study early in the process can also have disadvantages: the limited evidence strength (CFIR-construct, see Table 1) could have affected the motivation of involved settings, which could have led to implementation failures. No such failures were evident, as we were able to modify MeST in such a way that the core mechanism could be delivered feasibly and effectively in each RCP.

This study has several limitations. First, the process of implementation could have been more objective. In particular, attitudes of healthcare professionals were not assessed in a structured systematic way and we relied solely on feedback from staff and did not gather feedback from patients. Future studies might use focus groups of trainers of different settings, or patients in order to get standardized feedback of stakeholders. Patient engagement is critical to improving psychotherapy [41] and the next stage in investigations of the transportability of MeST must gather patient reports on acceptability and feasibility. In addition, for some settings we were unable to gather data on all patients given the additional burden this posed to clinicians. Such a limitation is to be expected when conducting research in RCPs. However, we nonetheless were able to gather data on 121 patients across seven settings.

Third, treatment fidelity and therapist competence were not formally assessed and no explicit supervision was provided. The authors were available for trainers when questions arose. It is possible, therefore, that the core component of specificity exercises was not delivered as prescribed in the manual. However the fact that comparable improvements in rAMS were achieved across settings under these conditions and relative to other MeST investigations [20] supports the suggestion that the intervention was delivered as intended. A fourth limitation regards selection bias. The seven RCPs all showed interest in MeST while the evidence strength was limited at that point. One can assume that the settings involved trusted the Intervention Source (CFIR-construct, see Table 1). It is unclear how this implementation effort would be similar for settings which dislike or have no interest in MeST for other reasons and might be more sceptical of the Intervention Source. For example, MeST can be regarded as an add-on intervention in the tradition of Cognitive Behavioural Therapy (CBT). Implementing MeST in settings who treat depressed patients with therapies from other traditions, such as psychodynamic psychotherapy, might pose different challenges. Subsequent studies might approach clinical settings randomly where no a priori knowledge of MeST or the Intervention Source is known. In addition, all settings involved shared cultural overlap as they are all from the Flemish region of Belgium. Cultural transportation is an important aspect of transportability [42] which future research should address. Another possible criticism on this study is that its main focus is on initial implementation whereas the sustainability of any implementation represents another important challenge within the implementation of interventions [24]. Nonetheless, three settings managed to continue an *open* version of MeST for several years, which is an indication that sustainable implementation of MeST seems possible. A last remark is that in all settings except one MeST was combined with the regular therapy program. The contribution of this interaction to the change in memory specificity and change in secondary outcomes might have varied over settings. For example, for the setting (with a small sample of five participants) where MeST was offered exclusively, no statistically significant impact of MeST on depressive symptomatology and brooding was found. MeST is not intended to be a standalone treatment for depression as it only targets one vulnerability factor. Future studies using bigger samples might examine interaction effects of MeST with other specific intervention characteristics in combination with specific patient characteristics such as diagnoses and age. Next possible steps in the dissemination and implementation of MeST are the use of rigorous study designs such as randomized clinical trials, to examine the effect of adaptable MeST in Routine Clinical Practices.

## Conclusions

MeST is transportable to RCPs such that it is feasible for those local clinicians delivering it and it continues to be as effective in RCPs as it is in research settings. In the present study, local needs of RCPs were met by dismantling MeST into different subparts. By dismantling it in this way, we were able to address several challenges raised by clinicians. In particular, multidisciplinary teams could divide the workload across different team members and, for the open version of MeST, the intervention could be offered continuously with tailored dosing per patient. Both closed and open versions of MeST, with or without peripheral components such as the STOP-model, and delivered by health professionals with different backgrounds, resulted in a significant increase in memory specificity for depressed in- and out- patients in RCPs. MeST may have beneficial effects not only in research settings but also in routine clinical settings where depressed people are most likely to access treatment.

## Additional file

Additional file 1: Raw data. (XLSX 27 kb)

## Abbreviations

AMT: Autobiographical Memory Test
BDI-II: Beck Depression Inventory II
CFIR: Consolidated Framework For Implementation Research
MeST: Memory Specificity training
OGM: Overgeneral Autobiographical Memory
PHQ-9: Patient Health Questionnaire 9
rAMS: Reduced Autobiographical Memory Specificity
RCP: Routine Clinical Practice
RRS-Brooding: The Ruminative Response Scale – Brooding subscale
